# Effect of breaking up prolonged sitting with physical activity on executive function: a three-level meta-analysis

**DOI:** 10.1186/s12966-026-01942-9

**Published:** 2026-06-23

**Authors:** Zan Huang, Mingyue Yin, Geng Li, Jinrong He, Qing Yi, Mingnan Zhuang, Bitai Wu, Meynard John Lapore Toledo, Waris Wongpipit, Wenxin Xu

**Affiliations:** 1 School of Physical Education and Sports Science, Fujian Normal University, No.18 Wulongjiang Middle Avenue, Shangjie Town, Minhou District, Fuzhou, China; 2 Guangdong Provincial Key Laboratory of Speed Capability Research, Jinan University, Guangzhou, China; 3 School of Psychology, Research Center for Exercise and Brain Science, Shanghai University of Sport, Shanghai, China; 4 School of Physical Education, Shanghai University of Sport, Shanghai, China; 5 Department of Physical Education, Tsinghua University, Beijing, China; 6 School of Sport Training, Tianjin University of Sport, Tianjin, China; 7 School of Human Movement Science, Hebei Sport University, Shijiazhuang, China; 8 School of Physical Education, Changsha University of Science and Technology, Changsha, Hunan China; 9 Center for Economic and Social Research, University of Southern California, Los Angeles, USA; 10 Division of Health and Physical Education, Faculty of Education, Chulalongkorn University, Bangkok, Thailand; 11 Center of Excellence in Educational Invention and Innovation, Faculty of Education, Chulalongkorn University, Bangkok, Thailand

**Keywords:** Executive function, Physical activity breaks, Physical activity, Cognitive performance, Meta-analysis

## Abstract

**Background:**

Prolonged sitting has been associated with adverse effects on cognitive function, and interrupting sitting with physical activity (PA) may help mitigate these effects. However, evidence regarding the effects of PA breaks on executive function remains limited. In addition, it remains unclear how intervention characteristics may be associated with variations in effect estimates. This study aims to synthesize evidence from randomized trials on the effects of PA breaks on executive function, and to identify potentially effective intervention characteristics through moderator analysis.

**Methods:**

A systematic search was conducted in Web of Science, PubMed, Embase, Scopus, Cochrane Library, APA PsycINFO, and SPORTDiscus from database inception to April 25, 2026, for randomized trials investigating the effects of PA breaks during sedentary time on executive function. Although the eligibility criteria allowed randomized controlled trials, randomized crossover trials, and cluster-randomized trials, all studies included in the final synthesis used randomized crossover designs. Risk of bias was assessed using the Cochrane Risk of Bias Tool version 2 for crossover trials, and the quality of evidence was evaluated using the Grading of Recommendations, Assessment, Development, and Evaluations framework. A random-effects three-level meta-analytic model was applied to analyze main effects, exploratory moderator effects, publication bias, and sensitivity analyses.

**Results:**

Twenty-two randomized crossover trials were included, comprising 123 effect sizes from 418 participants. Low certainty evidence suggests that, compared with uninterrupted sitting, PA breaks may be associated with a small improvement in executive function (Hedges’ *g* = 0.13, 95% CI [0.03, 0.23], *p* = 0.01), particularly working memory (Hedges’ *g* = 0.17, *p* = 0.02). Regrading performance metrics, reaction time (RT) performance (Hedges’ *g* = 0.18, *p* < 0.01) showed a significant positive effect, whereas accuracy showed no significant effect. Exploratory moderator analyses provided preliminary evidence that intervention characteristics may be related to variations in executive function outcomes. Light-intensity activities showed a favorable pooled estimate (Hedges’ *g* = 0.19, *p* < 0.01), while moderate-intensity activities did not show a significant pooled effect. For activity type, walking (Hedges’ *g* = 0.12, *p* = 0.03) and stair climbing (Hedges’ *g* = 0.70, *p* = 0.01) showed significant pooled effects, although the estimate for stair climbing was based on a small number of studies. Regarding frequency, 60-minute interruption intervals showed the largest pooled estimate (Hedges’ *g* = 0.30, *p* = 0.01). Break duration was not a statistically significant moderator (*p* = 0.06), although using 3–5 min breaks showed significant pooled effect (Hedges’ g = 0.16, *p* = 0.02).

**Conclusions:**

Interrupting prolonged sitting with PA is associated with small acute improvements in executive function, especially in the working memory and RT performance. Moderator analyses suggested that intervention characteristics such as activity intensity, type, and frequency may contribute to heterogeneity in effects. While the certainty of evidence is low and some subgroup estimates are based on a limited number of studies, these findings provide preliminary guidance for optimizing intervention design and highlight the need for further high-quality research.

**Trial registration:**

CRD420251004155.

**Graphical Abstract:**

**Figure:**
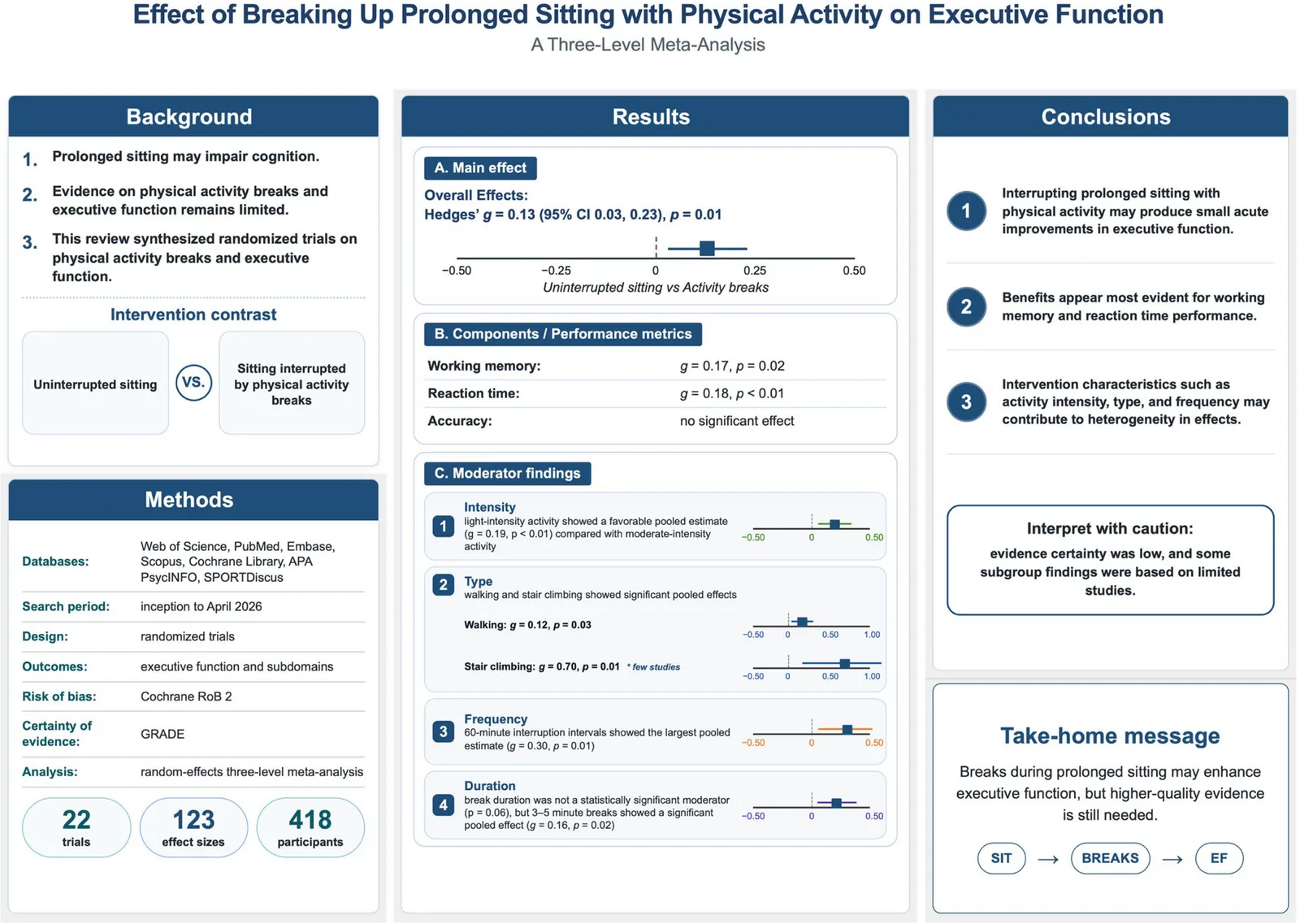

**Supplementary Information:**

The online version contains supplementary material available at https://doi.org/10.1186/s12966-026-01942-9.

## Background

Sedentary behavior, defined as any waking behavior characterized by an energy expenditure ≤ 1.5 metabolic equivalents (METs) while in a sitting or reclining posture [1], has become a global public health concern. People spend substantial time engaged in low-energy activities in seated or reclined postures during work, study, and leisure, such as using computers, watching television, or driving for extended periods [1]. Epidemiological studies have shown that sedentary behavior is significantly associated with increased risks of obesity, type 2 diabetes, cardiovascular disease, and all-cause mortality [2–4]. While the negative cardiometabolic effects of sedentary behavior are well-documented, consensus on its impact on cognitive function is still emerging [5]. Cognitive function refers to a broad set of mental processes that enable individuals to acquire, store, and use information, encompassing domains such as attention, memory, language, and executive function [6]. Executive function, as a higher-order aspect of cognitive function, involves the regulation of goal-directed thoughts and behaviors through three core components, including inhibitory control, cognitive flexibility, and working memory [7]. An increasing body of evidence indicates that prolonged sitting can lead to declines in attention, reductions in information-processing speed, and impairments in decision-making, which are closely associated with deteriorations in executive function [8].

Physical activity (PA) is widely recognized as a beneficial strategy for promoting cognitive health. Regular aerobic and resistance exercises can enhance executive function by improving cerebral blood flow, increasing neuroplasticity, and promoting the expression of neurotrophic factors such as brain-derived neurotrophic factor (BDNF) [9]. However, despite these benefits, traditional exercise interventions face practical barriers, including time constraints, lack of motivation, and occupational limitations [10]. As a feasible alternative strategy, breaking up prolonged sedentary periods with short bouts of PA has garnered increasing attention [11]. This approach involves periodic, brief, light-to-moderate intensity activities, such as standing, walking, or light resistance exercises, and does not require dedicated time for exercise and can be seamlessly integrated into daily routines [12, 13].

Several meta-analyses have examined the effects of breaking up sitting with PA on cognitive function. For example, Chueh et al. reviewed seven randomized trials and found that only three supported beneficial cognitive effects from moderate-to-vigorous PA during prolonged sitting [14]. Building on this, Li et al. [15] conducted the first quantitative meta-analysis, concluding that acute PA breaks during prolonged sitting did not affect global cognitive performance. In contrast, Feter et al. [16] systematically reviewed 25 randomized controlled trials and found that interrupting prolonged sitting multiple times with acute PA improved cognitive outcomes. A potential reason for these inconsistent findings is the aggregation of diverse cognitive tasks into a broad global cognitive function. This approach may dilute genuine, specific effects of PA breaks, particularly on executive function. Executive function is subserved by prefrontal brain regions that are highly sensitive to transient metabolic and vascular changes induced by PA [17]. It is plausible that breaking up sitting may produce more immediate and measurable benefits in this specific domain compared to other cognitive aspects. Therefore, it is important to specifically examine the impact of PA breaks on executive function. However, even when focusing on executive function, evidence remains mixed. Some studies observed significant improvements in subdomains such as inhibitory control and working memory [18, 19], whereas others did not find measurable benefits [20]. Beyond this heterogeneity in the findings, a more fundamental limitation is the lack of systematic investigation into how intervention parameters (i.e., the FITT-V framework: Frequency, Intensity, Type, Time, and Volume) moderate these effects. This gap severely limits the translation of findings into practical, optimized recommendations.

To address these issues, the present systematic review and three-level meta-analysis focused specifically on executive function. By synthesizing randomized trials and exploratorily examining whether FITT-V parameters moderate the effects of PA breaks, this review aimed to provide a more focused and cautious quantitative assessment of the effects of interrupting sitting with PA on executive function.

## Methods

### Registration

This study followed the Preferred Reporting Items for Systematic Reviews and Meta-Analyses (PRISMA) guidelines [21] and was registered in the PROSPERO database (Registration number: CRD420251004155). The PRISMA checklist is presented in Supplementary file Table S1.

### Literature search strategy

The search strategy was developed by two reviewers (ZH and MY) with experience in systematic reviews and meta-analyses, reviewed by the third reviewer (WX), and refined through pilot searches to ensure that key known studies were retrieved.

A comprehensive literature search was conducted in Web of Science Core Collection, PubMed/MEDLINE, Embase, Scopus, Cochrane Library, APA PsycINFO, and SPORTDiscus databases. The initial search included studies published up to February 20, 2025, and an updated research was conducted on April 25, 2026. Medical Subject Headings and Boolean operators were used to construct the search strategy targeting studies on PA breaks during sedentary time and executive function. Detailed search strategies for each database are provided in Supplementary file Table S2. Previous reviews and meta-analyses were also screened to identify additional references. Endnote 21 (Thompson ISI Research Soft, Philadelphia, PA, USA) was used for reference management and duplicate removal. After deduplication, records were screened using a combination of manual screening and the artificial intelligence-assisted screening tool ASReview. Two independent reviewers (ZH and MY) screened studies for eligibility based on a four-step process: (1) removal of duplicates, (2) exclusion based on title, (3) exclusion based on abstract, and (4) full-text screening. ASReview was used to support and prioritize the title and abstract screening process, while final inclusion and exclusion decisions were made independently by the reviewers according to the prespecified eligibility criteria. Disagreements were resolved by a third reviewer (WX).

### Study selection

Only peer-reviewed publications were included, with no restrictions on language or region. Grey literature and preprints were excluded. The inclusion criteria followed the Population, Intervention, Comparison, Outcome (PICOS) framework [22]: (1) Participants: Individuals without diagnosed mental or physical conditions affecting cognition or PA, with no age restrictions. (2) Intervention: Prolonged sitting was interrupted with multiple PA bouts, regardless of intensity and PA modes. This criterion was used to distinguish repeated sedentary-interruption strategies from single acute exercise exposures embedded within a sitting period. (3) Comparator: Uninterrupted sitting condition. (4) Outcomes: Executive function was measured in at least one of the three core executive function domains, namely working memory, inhibitory control, or cognitive flexibility, as defined by Diamond’s framework [7]. (5) Study Design: Randomized designs, including RCTs, randomized cross-over trials, or cluster-randomized trials. Although the eligibility criteria allowed several randomized designs, all studies ultimately included in the review used randomized crossover designs.

The following were excluded in the analysis: (1) Studies where PA effects could not be isolated from multi-component interventions; (2) Studies that do not report numerical outcome data sufficient for effect-size calculation (for example, means and standard deviations, change scores with measures of variability, standard errors, confidence intervals); (3) Study protocols or conference abstracts.

### Risk of bias assessment

Given that all included studies were randomized crossover trials, the Risk of Bias Tool for Crossover Trials version 2 (RoB 2), developed by Cochrane’s methodology group, was used [23]. The tool covers six domains: randomization process, period/carryover effects, deviations from intended interventions, missing outcome data, measurement of outcomes, and selection of the reported result. Two reviewers independently assessed each study. Ratings were classified as “low risk”, “some concerns”, or “high risk”, and disagreements were resolved through discussion. Results were presented in risk-of-bias graphs.

### Quality of evidence

The quality of evidence for all outcomes was assessed using the Grading of Recommendations Assessment, Development, and Evaluation (GRADE) framework, which evaluates certainty based on risk of bias, inconsistency, indirectness, imprecision, and publication bias [24]. Two reviewers conducted evaluations independently, with a third reviewer adjudicating disagreements.

### Data extraction

Two researchers independently extracted data into Excel, with cross-checking by a third reviewer. Extracted information included: first author, publication year, participant characteristics (e.g., age, sample size), PA breaks intervention details (e.g., frequency, type, intensity, and time), and outcome measures.

### Variable coding

Core variables were systematically coded to ensure consistent effect size calculations and the feasibility of subsequent moderator analyses. Based on the classical three-dimensional model, executive function was divided into three subdomains: working memory, inhibitory control, and cognitive flexibility. The specific task names and associated subdomains were recorded accordingly. For performance indicators, two types of outcomes were extracted: reaction time (RT) and accuracy. Since RT is a negatively oriented measure (i.e., smaller values indicate better performance), RT values were reverse-coded (i.e., multiplied by − 1) before effect size calculation to ensure that all positive effect sizes consistently represented performance improvement. For PA intervention parameters, the following were coded: intensity, type, frequency, break time, and total break-time volume. PA-break intensity was coded as light-intensity physical activity (LPA) or moderate-intensity physical activity (MPA) according to a hierarchical procedure based on the American College of Sports Medicine (ACSM) exercise-intensity criteria [25]. When intensity was explicitly reported by the original study authors, the reported classification was used. When intensity was not explicitly reported, available physiological or workload-related indicators were used, including oxygen uptake-based indicators such as oxygen uptake (VO₂), percentage of maximal oxygen uptake (%VO₂max), or VO₂ reserve; heart rate-based indicators such as heart rate (HR), percentage of maximal heart rate (%HRmax), or heart rate reserve (HRR); metabolic equivalents (METs); rating of perceived exertion (RPE); workload; walking speed; or equivalent exercise-intensity information. Light-intensity PA was generally defined as 1.6–2.9 METs, < 40% HRR or VO₂ reserve, < 64% HRmax, or RPE 9–11 on the Borg 6–20 scale, whereas moderate-intensity PA was generally defined as 3.0–5.9 METs, 40–59% HRR or VO₂ reserve, 64–76% HRmax, or RPE 12–13. When both oxygen uptake-based and heart rate-based indicators were available, oxygen uptake-based information was prioritized because it more directly reflects metabolic intensity. No included study used vigorous- or high-intensity PA breaks; therefore, vigorous intensity was not coded as a separate category. PA breaks type was coded according to the specific activity mode, including walking, stair climbing, resistance exercise, cycling, and yoga. Frequency was coded as once per hour, twice per hour, or more than twice per hour. Break time was coded as 1–2 min, 3–5 min, or ≥ 6 min. Total break time volume was calculated as a continuous variable to investigate its influence on treatment effects. For participant characteristics, we recorded age group, gender composition, and weight status. Age group was categorized as children/adolescents, young adults, middle-aged adults, or older adults; gender composition was categorized as female-only, male-only, or mixed; and weight status was classified as normal BMI range or overweight/obesity according to BMI-based criteria or the classifications reported in the original studies. Detailed coding information was provided in Supplementary file Table S3. All variables were independently coded by two researchers, with discrepancies resolved through discussion.

### Statistical analysis

#### Effect size calculation and aggregation

Data from each study were extracted, and effect sizes were calculated. Since studies often reported multiple outcome measures (e.g., across subdomains or performance metrics), it was possible to extract multiple effect sizes from a single study. These effect sizes have a nested structure (i.e., multiple outcome indicators within the same study). Conventional two-level meta-analytic models assume independence of effect sizes, which can lead to Type I errors [26]. Therefore, a three-level meta-analysis was employed using restricted maximum likelihood estimation to derive pooled effect sizes, 95% confidence intervals, and *p*-values. The three-level model accounted for three sources of variance: sampling variance (Level 1), within-study variance (Level 2), and between-study variance (Level 3) [27]. To verify the robustness of results, robust variance estimation (RVE) was performed after the three-level analysis. The three-level meta-analysis was conducted using the “*metafor*” package in R, and RVE was performed using the “*ClubSandwich*” package in R. Specifically, we implemented the more precise variance-covariance matrix approach combined with CR2 small-sample correction. This involved constructing study-specific variance-covariance matrices using an assumed within-study correlation of ρ = 0.5 between effect sizes, followed by application of CR2 correction to ensure robust inference under small-sample conditions.

Hedges’ g was used to estimate standardized effect sizes. Hedges’ *g* is a bias-corrected version of Cohen’s d that is more appropriate for small sample sizes [28]. Effect sizes were calculated using post-intervention values, which are recommended for randomized crossover trials over change-from-baseline values [29, 30]. When outcome data were presented only in graphical form, we used WebPlotDigitizer 4.1 (https://automeris.io/WebPlotDigitizer) to extract the necessary information. When required values were not available in the published articles, we contacted the authors. If no response was received, the study was excluded from the analysis.

#### Heterogeneity analysis

Cochran’s *Q* and *I²* statistics were used to assess heterogeneity. *Q* is defined as the weighted sum of squared differences; a *p*-value less than 0.1 indicates significant heterogeneity. *I²* quantifies the proportion of total variation that is due to heterogeneity rather than sampling error. In the three-level model, *I²* is partitioned into within-study (Level 2) and between-study (Level 3) components. Prediction intervals (PI) were also calculated to estimate the expected range of true effect sizes in future studies.

#### Moderator analysis

To examine whether participant characteristics and PA intervention features moderated the overall effect size, we conducted subgroup analysis and meta-regression using the three-level model. Moderator variables included participant characteristics (age, sex, and BMI) and FITT-V parameters (frequency, intensity, type, time, and volume). *F*-tests were used to assess significance. Consistent with the PROSPERO registration, age, baseline sedentary behavior, and study design were considered as potential moderators. Age was examined as a participant-level moderator. However, baseline sedentary behavior could not be examined because the included studies did not consistently report participants’ habitual sedentary time before the experimental session, and the available information was insufficient for meaningful comparison across studies. Study design was also not examined as a moderator because all studies included in the final synthesis used randomized crossover designs, leaving no between-study variation in design.

#### Sensitivity analysis

To evaluate the robustness of the pooled effect size and the reliability of results, multiple sensitivity analyses were conducted. First, influential effect sizes were identified using diagnostic measures such as standardized deleted residuals and Cook’s distance [31, 32]. We compared models before and after excluding influential cases to assess changes in effect size estimates, direction, and heterogeneity indices (e.g., *I²*) [33]. Second, we performed leave-one-out analysis, in which each study was excluded one at a time and the pooled effect size recalculated to determine the influence of individual studies. Third, forest plots were used to visually inspect the distribution and confidence intervals of effect sizes, helping to identify potential outliers and assess heterogeneity without introducing subjective bias [34]. Finally, we assessed the impact of different within-study correlation assumptions (0.3, 0.6, and 0.8) used in the RVE, confirming that results remained stable across this range of plausible correlation values.

#### Publication bias

Funnel plot and Egger’s regression tests were used to detect potential publication bias, conducted at both Level 2 (within-study variance) and Level 3 (between-study variance). Symmetrical funnel plots and non-significant Egger’s tests (*p* > 0.05) indicated no substantial publication bias [35]. If bias was detected, the trim-and-fill method was used for further verification [36].

To assess the potential for selective reporting (*p*-hacking) and evaluate the evidential value of statistically significant findings, we conducted p-curve analysis following the procedures described by Simonsohn et al. [37]. All *p*-values below 0.05 related to the primary hypothesis were extracted and converted to *Z*-scores. Full and half *p*-curves were tested for right-skewness using binomial and *Z*-tests (with 5,000 Monte Carlo simulations). The presence of significant right-skewness supported the existence of evidential value, ruling out selective reporting as the sole explanation for positive findings. Flatness tests were also conducted to check for inadequate statistical power. The overall statistical power was estimated from the observed p-value distribution.

## Results

### Search results

**Fig. 1:**
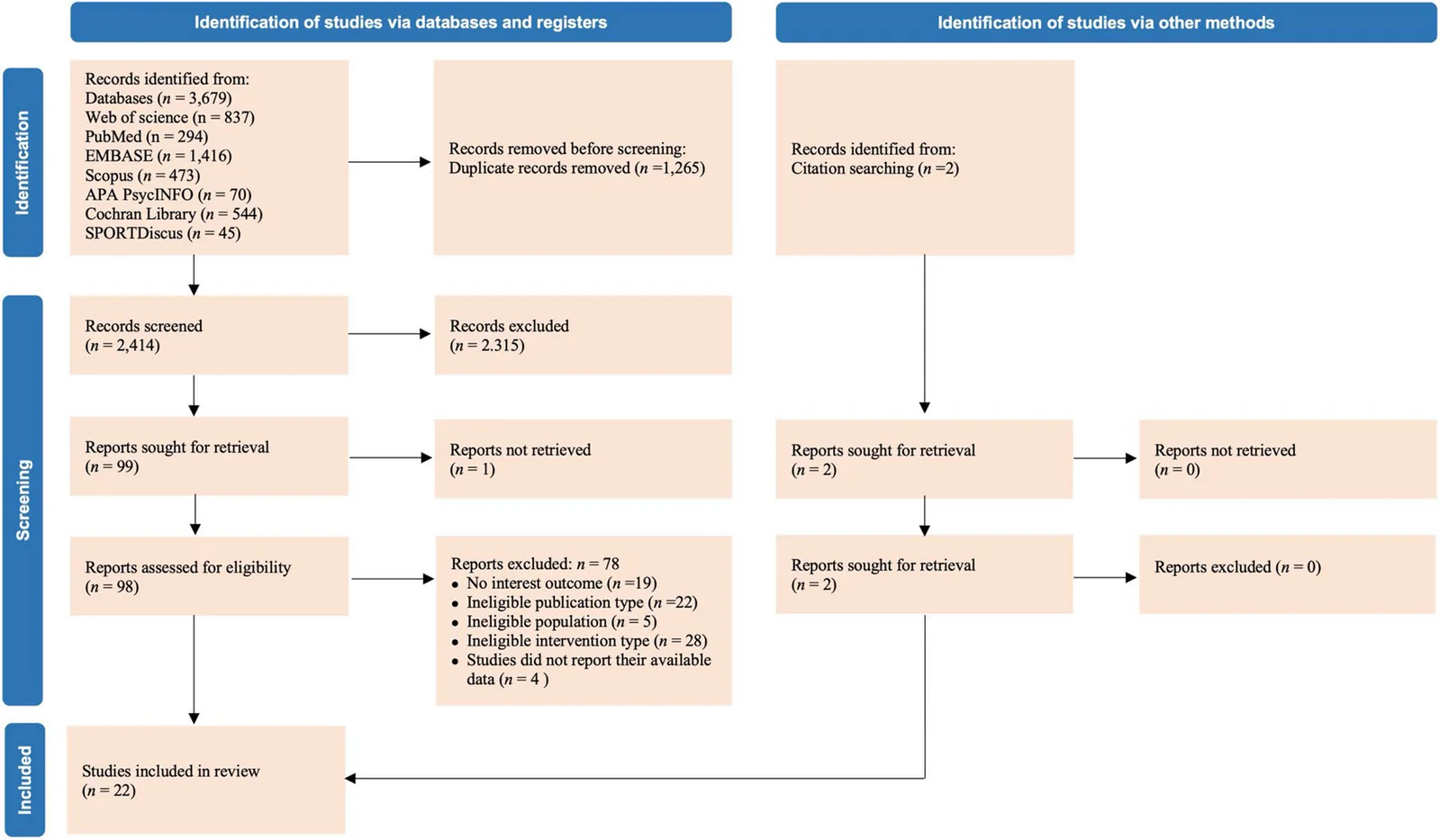
PRISMA Flow diagram

The PRISMA flow diagram (Fig. 1) illustrates the study selection process. The initial search identified 3,679 potential studies. After removing duplicates, 2,414 unique records remained. Titles and abstracts were screened by two independent reviewers, yielding 98 articles for full-text review. Following full-text evaluation, 20 studies met the inclusion and exclusion criteria. Moreover, two studies were retrieved form citations searching. Finally, 22 studies included in the review.

### Study characteristics

Table 1 summarizes the characteristics of the included studies. A total of 22 randomized crossover trials were included [18–20, 38–56], published between 2016 and 2025, with a combined sample of 418 participants aged between 13.6 and 78 years. Across the included studies, PA breaks protocols involved walking, resistance exercise, cycling, and stair climbing. Type counts were not mutually exclusive, as studies with multiple eligible intervention arms or activity types could contribute to more than one category. These counts therefore refer to study-level classifications rather than mutually exclusive groups. Walking was represented in 17 studies [18–20, 38, 39, 41–44, 47, 48, 50, 52–56], resistance exercise in six studies [18, 43, 45, 48, 49, 51], cycling in two studies [47, 53], stair climbing in one study [46], and yoga in one study [40], with some studies investigating multiple intervention types [18, 43, 46, 48, 53]. The duration of PA breaks per session ranged from 1 to 30 min. Break intervals varied from 10 to 60 min. Sedentary control conditions in uninterrupted sitting ranged from 1 h 20 min to 13.5 h of uninterrupted sitting. Most studies assessed only one executive function subdomain [18, 19, 38, 39, 42–44, 46–53, 55], and only six evaluated two or more [20, 40, 41, 45, 54, 56]. In total, 13 studies assessed inhibitory control [20, 39–46, 51, 52, 55, 56], 10 assessed working memory [18–20, 38, 40, 47–50, 53], and 5 assessed cognitive flexibility [20, 41, 45, 54, 56]. All executive function tests were conducted immediately following the intervention.

**Table 1:** Characteristics of the included studies

Study	Country	*N*	Females (%)	Age (years)	Intervention arm	Control arm	Executive domain	Test time	Instrument
Bergouignan et al. 2016 [56]	Sweden	30	43	30.0 ± 5.6	Treadmill walking, 6 × 5-min MPA bouts every 60 min; RPE 12–13	Uninterrupted sitting 6 h 20 min	Inhibitory control Cognitive flexibility	Immediately after intervention	Flanker task TMT
Chandran et al. 2023 [46]	India	21	19	26.7 ± 2.6	Arm 1: Walking, 4 × 3-min LPA bouts every 60 min Arm 2: Stair climbing, 4 × 3-min MPA bouts every 60 min	Uninterrupted sitting 4 h	Inhibitory control	Immediately after intervention	Flanker task
Chrismas et al. 2019 [52]	Qatar	11	100	21–44	Treadmill walking, 9 × 3-min MPA bouts every 30 min; RPE 12–14	Uninterrupted sitting 5 h	Inhibitory control	Immediately after intervention	Stroop task
Heiland et al. 2021 [48]	Sweden	13	38	50.5 ± 4.6	Arm 1: Treadmill walking, 6 × 3-min MPA bouts every 30 min; 75–80% HRmax Arm 2: Resistance exercise, 6 × 3-min MPA bouts every 30 min	Uninterrupted sitting 3 h	Working memory	Immediately after intervention	N-back task
Maasakkers et al. 2020 [50]	Netherlands	22	41	78.0 ± 5.3	Arm 1: Walking, 6 × 2-min LPA bouts every 30 min Arm 2: Walking, 6 × 2-min LPA bouts every 30 min	Uninterrupted sitting 3 h	Working memory	Immediately after intervention	TAP
Mullane et al. 2017 [53]	USA	9	78	30.0 ± 15.0	Arm 1: walking, 8 LPA bouts: 2 × 10 min, 2 × 15 min, 2 × 20 min, and 2 × 30 min at 1.6 km/h; every hour between 0850–1530 h Arm 2: Cycling, 8 LPA bouts: 2 × 10 min, 2 × 15 min, 2 × 20 min, and 2 × 30 min at 20 W, 25–30 rpm; every hour between 0850–1530 h	Uninterrupted sitting 8 h	Working memory	Immediately after intervention	N-back task
Wanders et al. 2020 [47]	Australia	24	79	59.6 ± 8.1	Cycling, 6 × 5-min MPA bouts every 30 min; 50–70% of estimated HRmax	Uninterrupted sitting 4 h	Working memory	Immediately after intervention	TAP
Wennberg et al. 2016 [55]	Australia	19	47	59.7 ± 8.1	Treadmill walking, 10 × 3-min LPA bouts every 30 min	Uninterrupted sitting 5 h	Inhibitory control	Immediately after intervention	Flanker task Stroop task
Wheeler et al. 2019 [19]	Australia	12	17	70.0 ± 7.0	Walking, 13 × 3-min LPA bouts every 30 min; RPE 9–11	Uninterrupted Sitting 8 h	Working memory	Immediately after intervention	N-back task
Wu et al. 2023 [20]	Netherlands	21	71	24.4 ± 4.0	Walking, 8 × 5-min LPA bouts every 25 min	Uninterrupted sitting 4 h	Inhibitory control Cognitive Flexibility Working memory	Immediately after intervention	Flanker task Stroop task TMT
Horiuchi et al. 2023 [45]	Japan	20	45	21.0 ± 1.0	Resistance exercise, 9 × 1-min LPA bouts every 20 min	Uninterrupted sitting 3 h	Inhibitory control Cognitive Flexibility	Immediately after intervention	Stroop task TMT
Duvivier et al. 2024 [54]	Netherlands	25	48	64.0 ± 7.0	Walking, 3 × 5-min LPA bouts every 30 min	Uninterrupted Sitting 13.5 h	Cognitive Flexibility	Immediately after intervention	TMT
da Silva et al. 2024 [43]	Brazil	12	66.6	28.0 ± 9.0	Arm 1: Walking, 6 × 2-min LPA bouts every 30 min Arm 2: Resistance exercise, 6 × 3-min LPA bouts every 30 min; 30% of maximal voluntary contraction	Uninterrupted sitting 3 h	Inhibitory control	Immediately after intervention	Flanker task Stroop task
Kuo et al. 2024 [44]	China	24	49	31.0 ± 8.0	Treadmill walking, 15 × 2-min MPA bouts every 20 min; RPE 10	Uninterrupted sitting 5.3 h	Inhibitory control	Immediately after intervention	Flanker task
Kjellenberg et al. 2024 [18]	Sweden	17	64.7	13.6	Arm 1: Resistance exercise, 4 × 3-min LPA bouts every 17 min Arm 2: Walking, 4 × 3-min LPA bouts every 17 min	Uninterrupted sitting 1 h 20 min	Working memory	Immediately after intervention	N-back task
Pindus et al. 2025 [38]	USA	19	63	29.7 ± 7.35	Walking, 5 × 3.5-min MPA bouts every 30 min; 54.2% heart rate reserve	Uninterrupted sitting 3 h	Working memory	Immediately after intervention	N-back task
Chueh et al. 2025 [42]	China	18	0	25.0 ± 4.0	Walking, 5 × 3-min MPA bouts every 30 min; RPE 9.4	Uninterrupted sitting 3.5 h	Inhibitory control	Immediately after intervention	Stroop task
Kuang et al. 2025 [39]	USA	19	63	29.9 ± 7.5	Walking, 5 × 3.5-min MPA bouts every 30 min; 55% heart rate reserve	Uninterrupted sitting 3 h	Inhibitory control	Immediately after intervention	Flanker task
Cunha et al. 2025 [41]	Brazil	27	92	69.4 ± 5.6	Walking, 6 × 2-min LPA bouts every 30 min	Uninterrupted sitting 3 h	Inhibitory control Cognitive Flexibility	Immediately after intervention	Stroop task TMT
Charlett et al. 2021 [49]	UK	20		25 ± 6	Resistance, 8 × 3 min MPA bouts every 30 min	Uninterrupted sitting 5 h	Working memory	Immediately after intervention	Probe memory test
Kamath et al.2025 [40]	India	15	42	30.24 ± 3.68	Arm 1: Pranayama, 4 × 3-min LPA bouts every hour Arm 2: Yoga, 4 × 3-min MPA bouts every hour	Uninterrupted sitting 4 h	Inhibitory control Working memory	Immediately after intervention	Flanker task N-back task
Stoner et al.2019 [51]	USA	20	70	21.7 ± 2.5	Resistance, 18 × 1-min LPA bouts every 10 min	Uninterrupted sitting 3 h	Inhibitory control	Immediately after intervention	Stroop task

Note: *LPA* Light-Intensity Physical Activity, *MPA* Moderate-Intensity Physical Activity, *TMT* Trail Making Test, *TAP* Test of Attentional Performance, *HRmax* Maximum Heart Rate

### Risk of bias

**Fig. 2:**
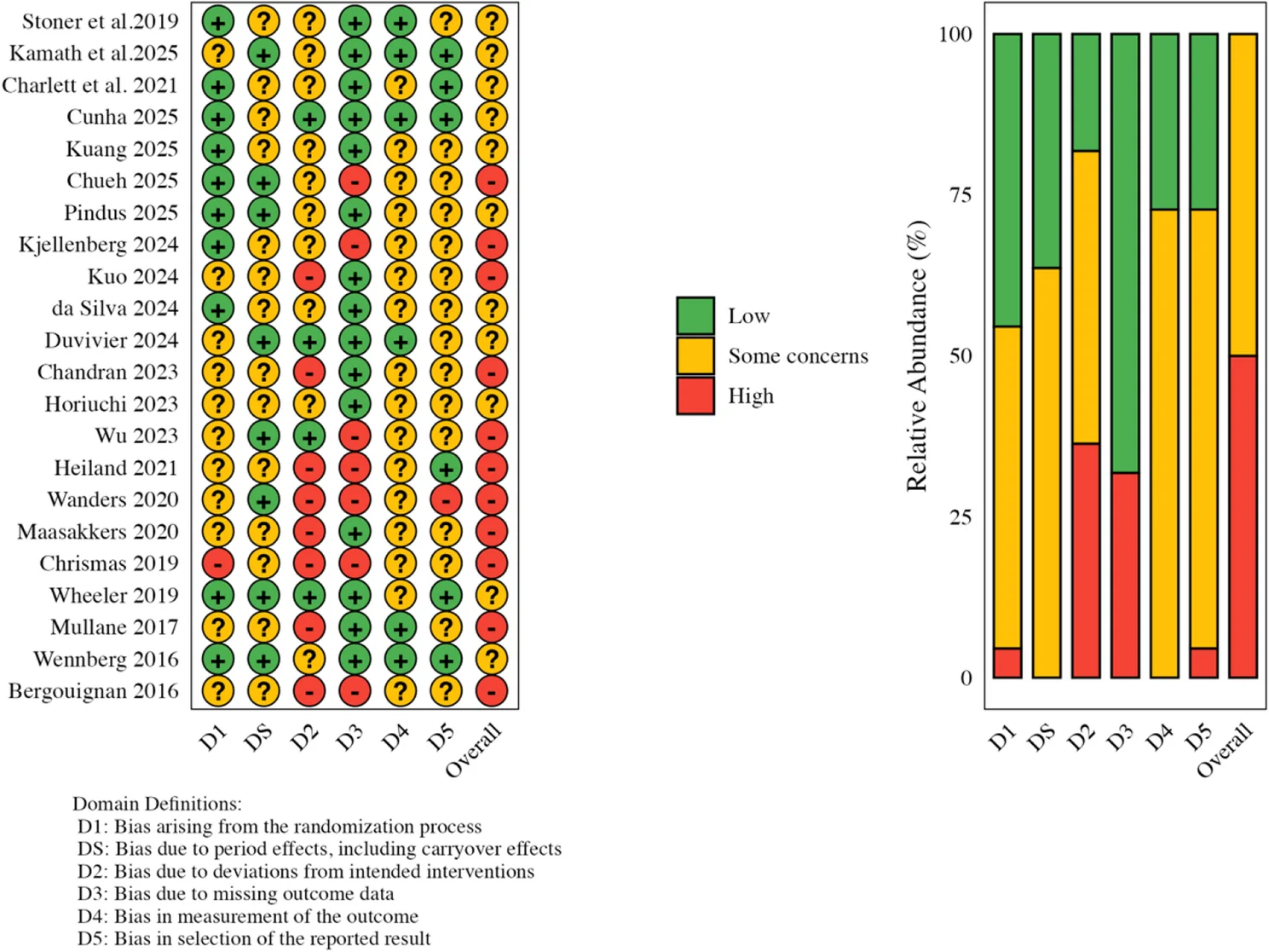
Results of risk of bias

Risk of bias was assessed using the RoB 2 for Crossover Trials tool (Fig. 2). For the randomization process, 45% (*n* = 10) of studies were rated low risk [18, 19, 38, 39, 41–43, 49, 51, 55], 50% (*n* = 11) raised some concerns due to insufficient description of random sequence generation or allocation concealment [20, 40, 43, 45–48, 50, 53, 54, 56], and 5% (*n* = 1) were high risk [52]. For carryover effects, 36% (*n* = 8) of studies were low risk [19, 20, 38, 40, 42, 47, 54, 55], 64% (*n* = 14) were of some concern [18, 39, 41, 43–46, 48–53, 56], and no study was high risk. Regarding deviations from intended interventions, 36% (*n* = 8) were high risk [44, 46–48, 50, 52, 53, 56], primarily due to limitations in implementing blinding, which may have introduced subjective bias in efficacy assessments, while 18% (*n* = 4) were low risk due to strict protocol adherence [19, 20, 41, 54]. The remaining 46% (*n* = 10) were rated as some concerns [18, 38–40, 42, 43, 45, 49, 51, 55], as insufficient information was reported. For missing data, 68% (*n* = 15) were low risk due to complete outcome reporting and dropout rates below 15% [19, 38–41, 43–46, 49–51, 53–55], while 32% (*n* = 7) were high risk due to the absence of intention-to-treat analysis or unclear missing data mechanisms [18, 20, 42, 47, 48, 52, 56]. Regarding outcome measurement, 73% (*n* = 16) were rated as having some concerns [18–20, 38, 39, 42–50, 52, 56], due to lack of standardized measurement tools or insufficient reporting of assessor training; 27% (*n* = 6) of studies were low risk [40, 41, 51, 53–55]. For selective reporting, 68% (*n* = 15) were rated as having some concerns [18, 20, 38, 39, 42–46, 50–54, 56], due to lack of pre-registration or potential selective reporting of positive findings, and 27% (*n* = 6) were rated low risk due to full data transparency [19, 40, 41, 48, 49, 55]. Overall, 50% (*n* = 11) of studies were judged high risk [18, 20, 42, 44, 46–48, 50, 52, 53, 56], due to cumulative concerns across domains, particularly carryover effects and blinding, while 50% (*n* = 11) were rated as having some concerns [19, 38–41, 43, 45, 49, 51, 54, 55].

### Main effects

**Fig. 3:**
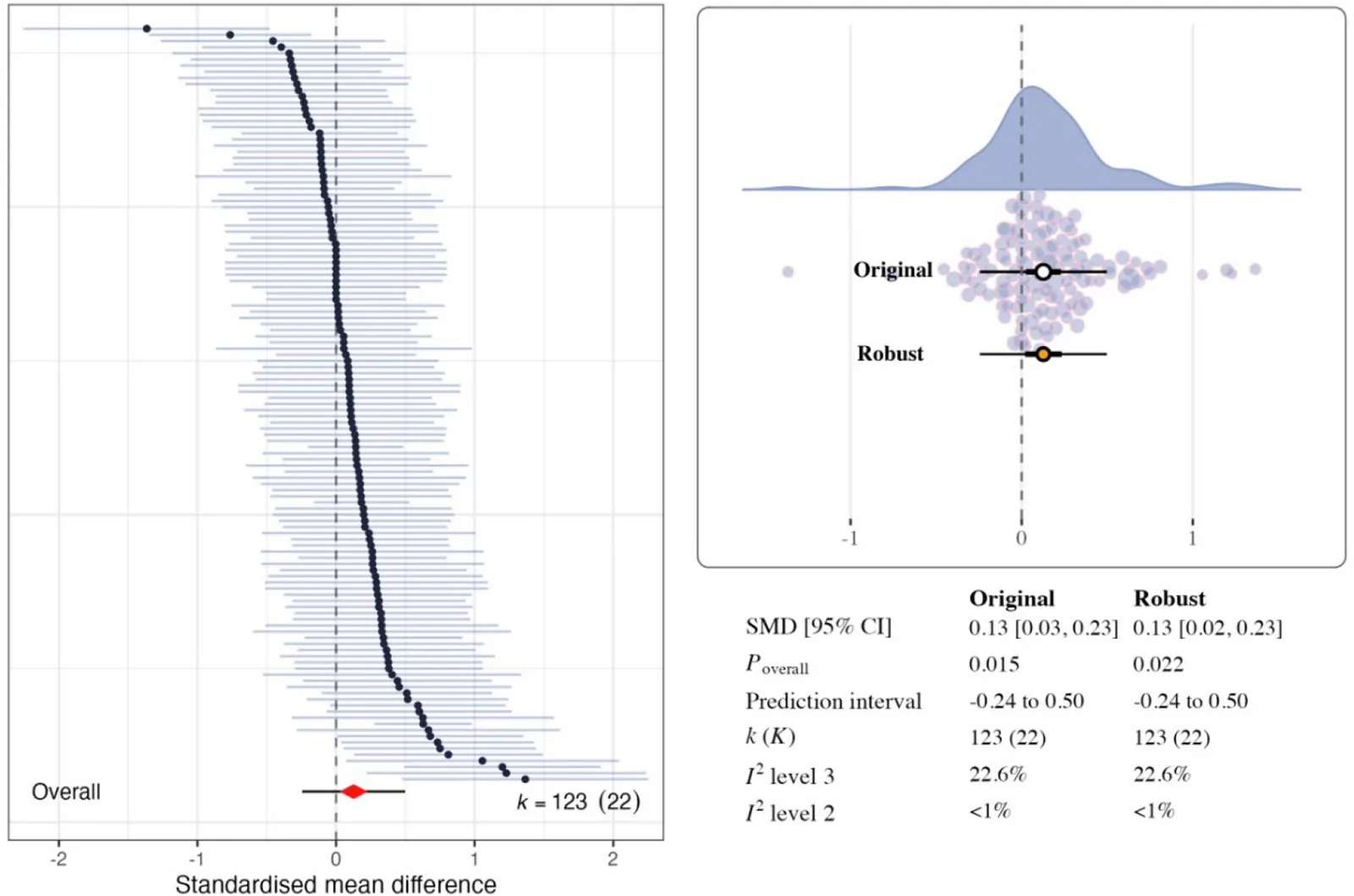
Overall effects of breaking up prolonged sitting with physical activity on executive function

**Fig. 4:**
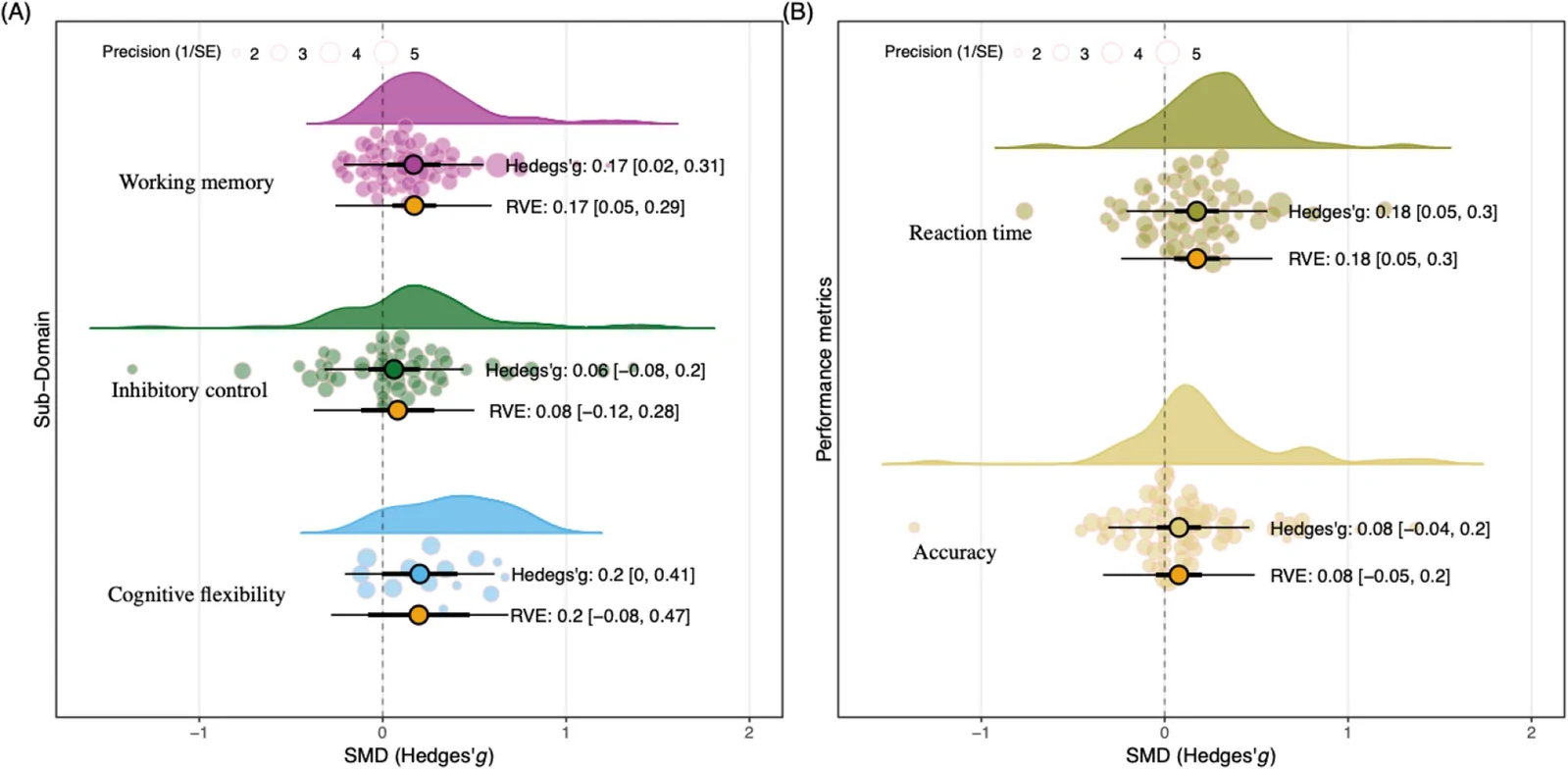
Sub-domain and performance metrics effects of breaking up prolonged sitting with physical activity on executive function

Interrupting sedentary behavior with PA showed a small but significant positive effect (Fig. 3) on global executive function (Hedges’ *g* = 0.13, 95% CI [0.03, 0.23], *p* = 0.01, PI = − 0.24 to 0.50). For subdomains (Fig. 4), working memory showed a significant effect (Hedges’ *g* = 0.17, 95% CI [0.02, 0.31], *p* = 0.02, PI = − 0.24 to 0.59), whereas cognitive flexibility (Hedges’ *g* = 0.20, 95% CI [− 0.01, 0.41], *p* = 0.06, PI = − 0.24 to 0.64) and inhibitory control (Hedges’ *g* = 0.06, 95% CI [− 0.08, 0.20], *p* = 0.39, PI = − 0.33 to 0.49) were not statistically significant. Regarding performance metrics, RT showed significant improvement (Hedges’ *g* = 0.18, 95% CI [0.05, 0.30], *p* < 0.01, PI = − 0.21 to 0.56), whereas accuracy did not show a significant change (Hedges’ *g* = 0.08, 95% CI [− 0.04, 0.20], *p* = 0.21, PI = − 0.31 to 0.46). The results remained robust after applying RVE.

Heterogeneity analysis indicated non-significant overall heterogeneity (*Q* = 110.90, *p* = 0.75), with no within-study heterogeneity (*I²*_level2_ = < 1%) and 22.56% of total heterogeneity attributed to between-study differences (*I²*_level3_ = 22.56%).

### Moderator Effects

**Fig. 5:**
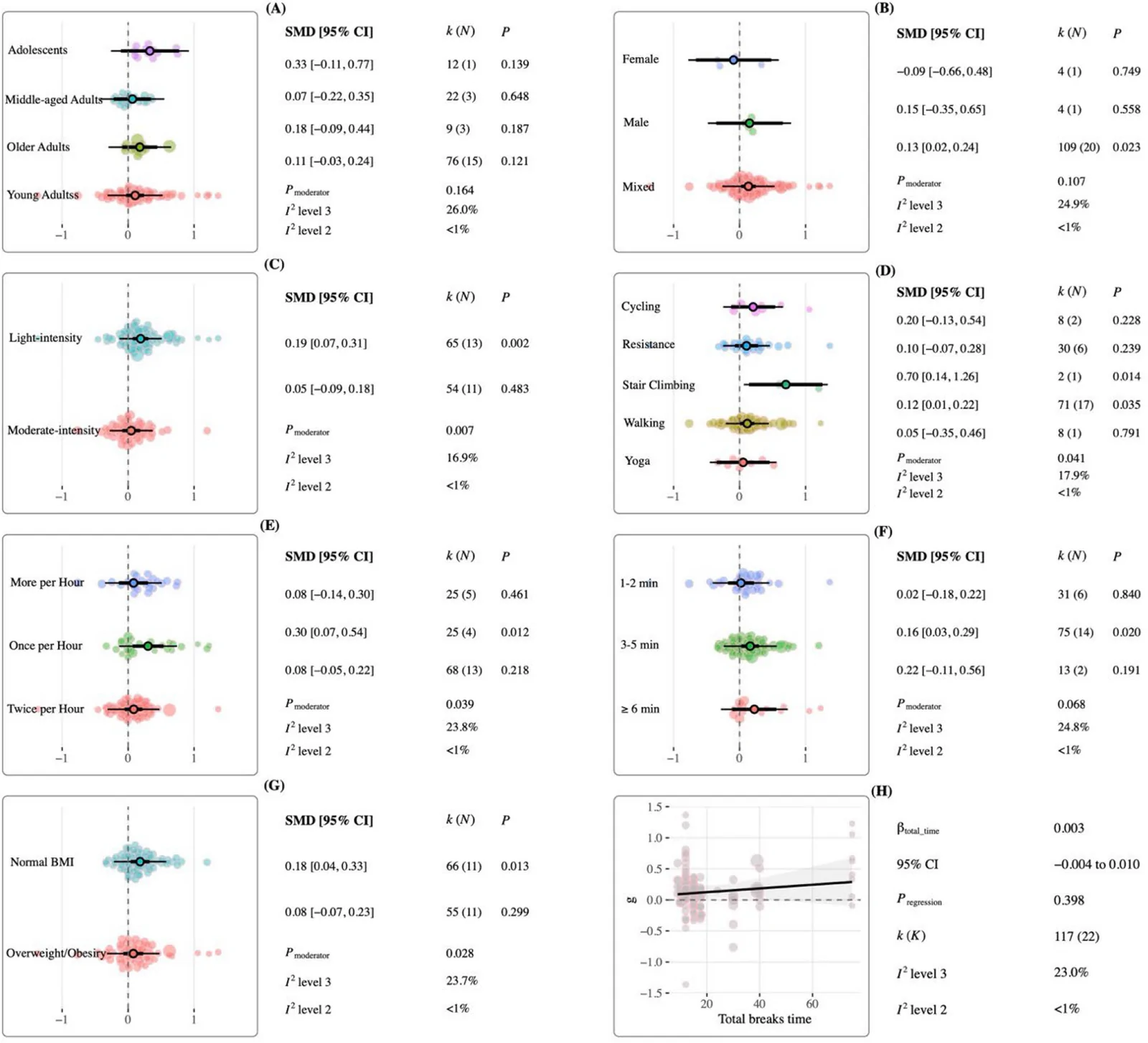
Moderator effects. Note: CI, Confidence Interval; *k*, number of effect sizes; *N*, number of studies. A, age; B, sex; C, intensity; D, type; E, frequency; F, time; G, BMI; H, volume

Subgroup analysis (Fig. 5) showed that age and sex did not significantly moderate the effect of PA breaks on executive function (*P*_Moderator_ > 0.05). However, BMI status did moderate the effect: significant benefits were found for participants with normal BMI (Hedges’ *g* = 0.18, 95% CI [0.04, 0.33], *p* = 0.01). Further moderator analyses of PA parameters showed that light-intensity activities significantly enhanced executive function (Hedges’ *g* = 0.19, 95% CI [0.07, 0.31], *p* < 0.01). Both stair climbing (Hedges’ *g* = 0.70, 95% CI [0.14, 1.26], *p* = 0.01) and walking (Hedges’ *g* = 0.12, 95% CI [0.01, 0.22], *p* = 0.03) were effective types. However, given the small number of stair-climbing effect sizes (*k* = 2), results should be interpreted with caution. A frequency of one break every 60 min significantly moderated the effects (Hedges’ *g* = 0.30, 95% CI [0.07, 0.54], *p* = 0.01). Moreover, the moderating effect of activity duration was nearly significant (*P*_Moderator_ = 0.06), with the effect size for 3–5 min breaks being significant (Hedges’ *g* = 0.16, 95% CI [0.03, 0.29], *p* = 0.02). To address the potential confounding between break duration and frequency, we further examined the moderating effect of total break time (duration × frequency). The analysis revealed that total break time volume was not a significant moderator (*b* = 0.003, 95% CI [–0.005, 0.010], *p* = 0.40), suggesting that the benefits are not driven by the cumulative volume of activity.

### Sensitivity analysis

Sensitivity analyses confirmed the robustness of results (Table 2). First, two outlier cases [18, 44] were identified (Supplementary file Figure S1). Excluding these cases slightly reduced heterogeneity (Hedges’ *g* = 0.13, 95% CI [0.04, 0.22], PI = − 0.15 to 0.41, *p* < 0.01, *I²*_*level2*_ = < 1%, *I²*_*level3*_ = 13.79%) without substantially altering effect sizes or direction. Leave-one-out analysis (Supplementary file Figure S2 and Table S4) showed minimal variation in the pooled effect sizes (maximum effect size: Hedges’*g* = 0.15, 95%CI [0.06, 0.24], minimum effect size: Hedges’*g* = 0.10, 95%CI [0.02, 0.18]). Forest plots (Supplementary file Figure S3 and Figure S4) showed relatively stable distributions across studies with no major anomalies. Additionally, RVE with different within-study correlation assumptions (ρ = 0.3, ρ = 0.6 and ρ = 0.8) yielded consistent positive effects (Hedges’ *g* = 0.11–0.12), with only minor variations in statistical significance and heterogeneity estimates. The stability of results across these diverse sensitivity checks supports the reliability of our primary findings.

**Table 2:** Sensitivity analysis results

Analysis Type	Hedges’ g [95% CI]	*p*-value	Q-statistic	I²_Level2_	I²_Level3_	Prediction Interval
Main Analysis	0.13 [0.02, 0.23]	0.01	110.90	< 1%	22.56%	[–0.24, 0.50]
Outlier Exclusion	0.13 [0.04, 0.22]	< 0.01	94.26	< 1%	13.79%	[–0.15, 0.41]
RVE (ρ = 0.3)	0.12 [0.02, 0.22]	0.03	112.04	< 1%	7.48%	[–0.10, 0.35]
RVE (ρ = 0.6)	0.12 [0.01, 0.22]	0.04	167.94**	< 1%	10.6%	[–0.14, 0.49]
RVE (ρ = 0.8)	0.11 [0.01, 0.22]	0.04	313.22***	< 1%	27.01%	[–0.33, 0.56]

Note: RVE, Robust Variance Estimation; CI represents the confidence interval; PI represents the prediction interval; **p < 0.01; *** *p* < 0.001

### Publication bias

**Fig. 6:**
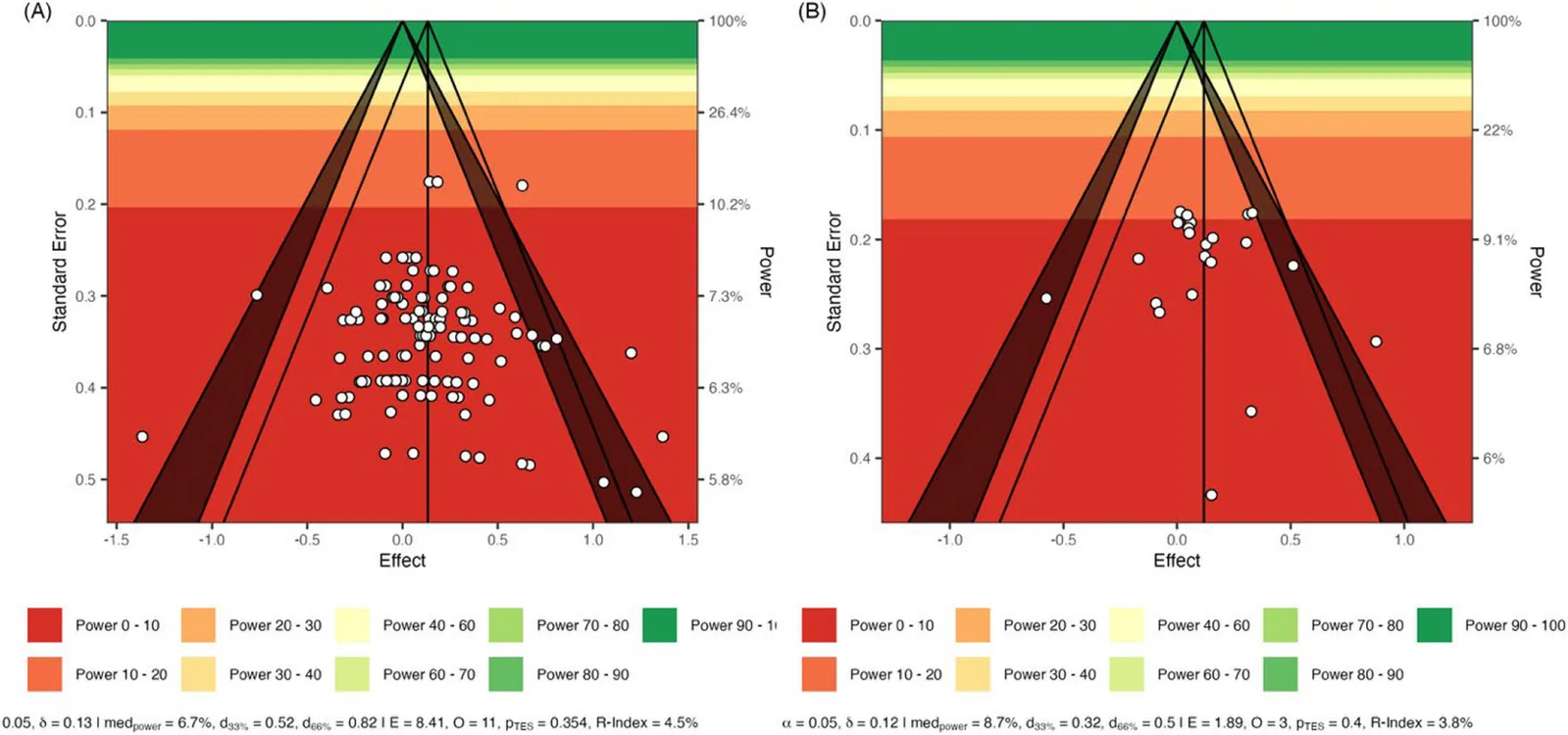
Funnel plots

Funnel plots and Egger’s regression tests assessed publication bias at both the within- and between-study levels (Fig. 6). The two-level funnel plot showed slight asymmetry, suggesting possible bias, but Egger’s test indicated no significant bias (*t*_(20)_ = 0.185, *p* = 0.855). The three-level funnel plot also showed no significant bias according to Egger’s regression (*t*_(121)_ = 0.504, *p* = 0.479).

**Fig. 7:**
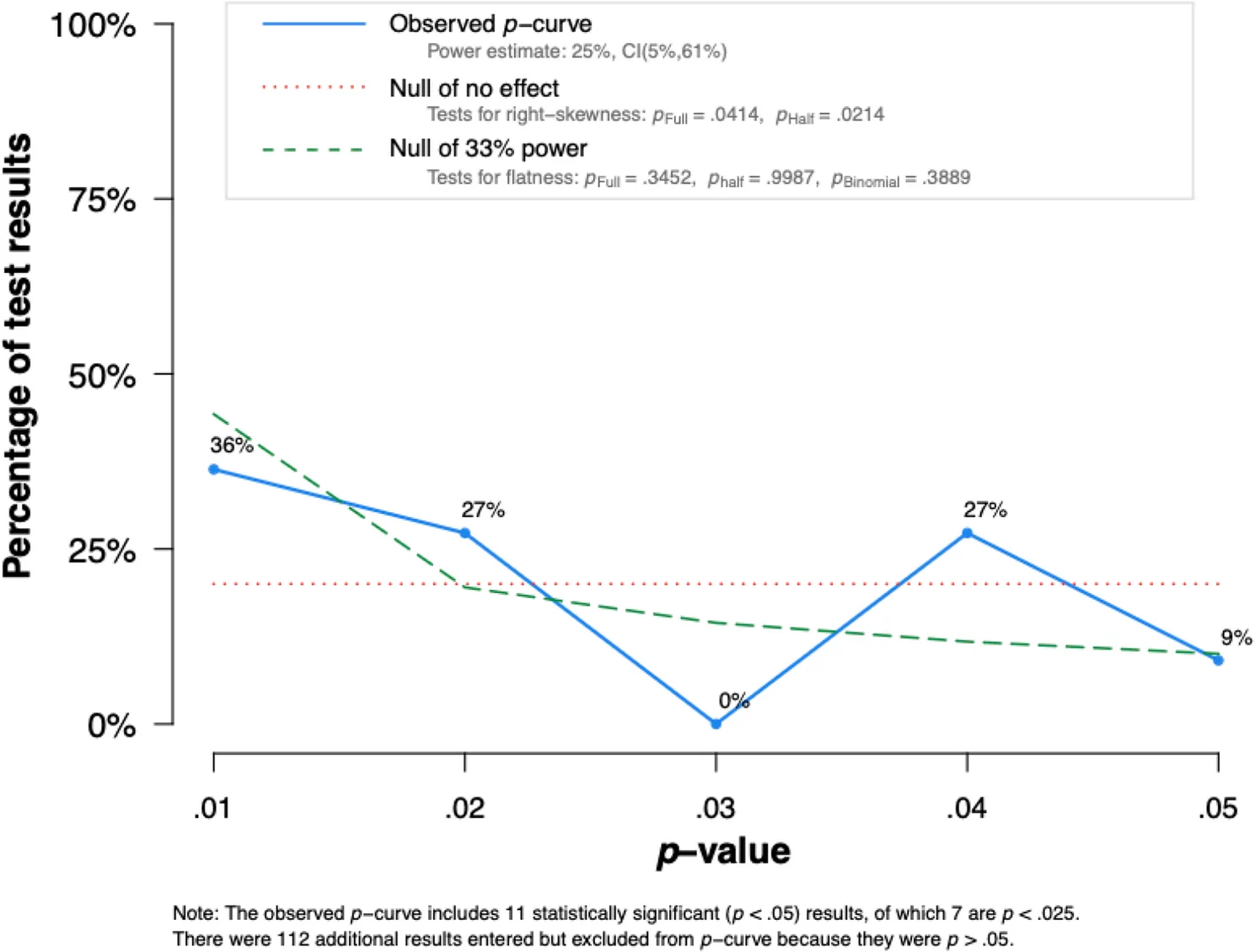
P‑curve analysis

A *p*-curve analysis of the 123 effect sizes found that 11 (8.94%) had *p*-values < 0.05 and 7 (5.69%) had *p*-values < 0.025. Binomial test (*p* = 0.274), full p-curve z-test (*z* = − 1.735, *p* = 0.041), and half p-curve z-test (*z* = − 2.026, *p* = 0.021) indicated a significant right-skew, supporting the evidential value of the observed results. Flatness tests (full curve *z* = − 0.398, *p* = 0.345; half curve *z* = 3.022, *p* = 0.999) did not indicate a flat distribution, ruling out insufficient power as a source of bias. Estimated statistical power was 25% (95% CI = 5.3%–60.8%), suggesting limited power but with confidence intervals crossing the 33% threshold, leaving some uncertainty. Overall, the right-skewed and non-flat *p*-curve supports the presence of true effects (Fig. 7).

### GRADE assessment

**Fig. 8:**
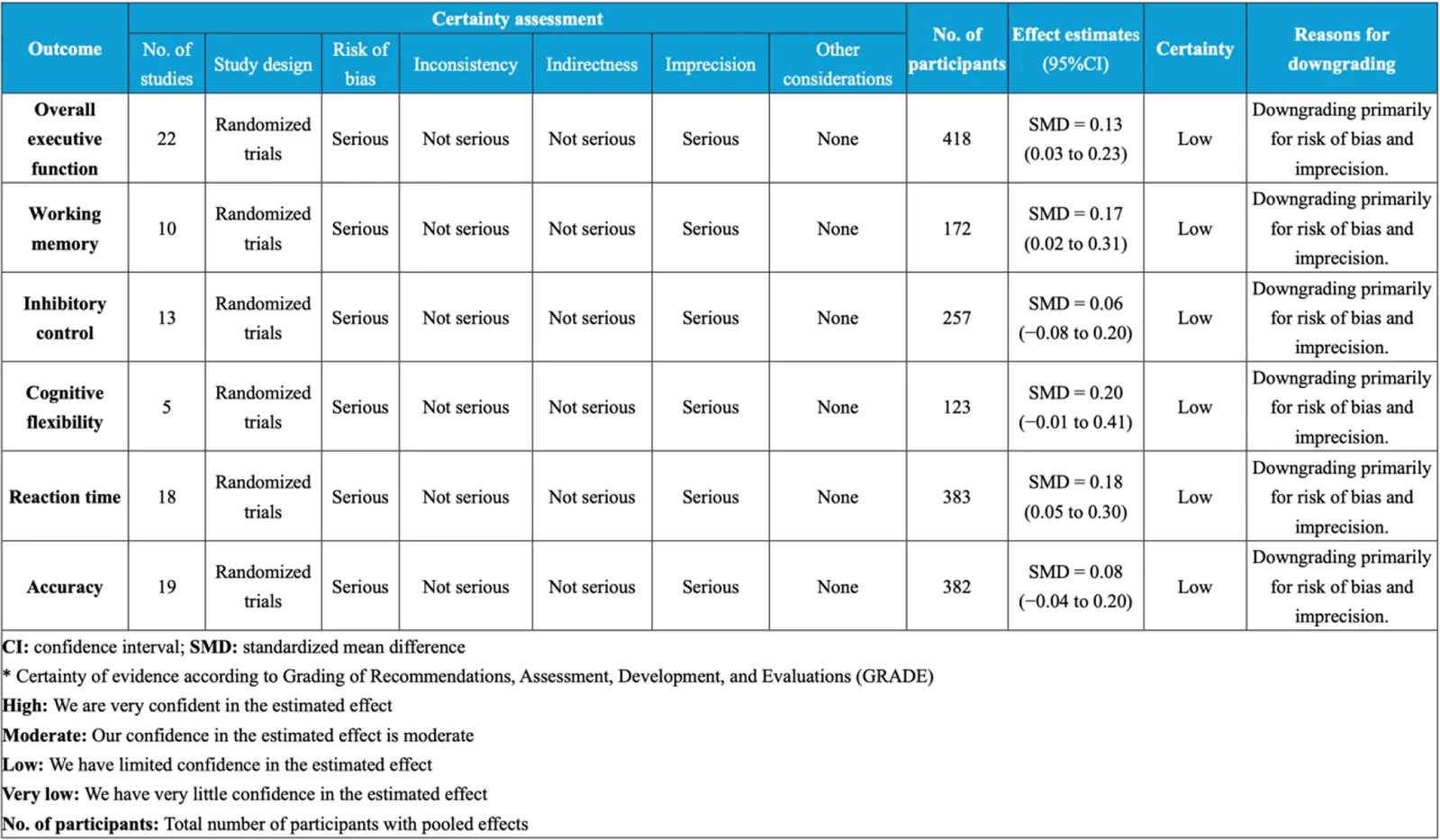
GRADE Assessment

The GRADE assessment results (Fig. 8) evaluated the quality of evidence for each outcome. All outcomes were rated as low quality due to methodological limitations, inconsistency, and risk of bias concerns.

## Discussion

This three-level meta-analysis synthesized randomized trials examining the acute effects of PA breaks on executive function. PA breaks demonstrated a small but statistically significant improvement in executive function compared with uninterrupted sitting. However, these results should be interpreted cautiously, given the low certainty of evidence and the variability in effects across executive function domains, performance indicators, and intervention characteristics. Overall, this review complements previous systematic reviews by providing an updated quantitative synthesis of randomized trials and an exploratory examination of how intervention characteristics may relate to variations in executive function outcomes.

Previous reviews on PA breaks and cognitive outcomes have reported mixed findings. Li et al. [15] conducted the first meta-analysis on this topic and found no significant effects of PA breaks during prolonged sitting on global cognitive function or its subdomains (e.g., attention, memory, executive function). More recently, another review further summarized evidence on PA breaks and cognitive outcomes, but questions remained regarding whether specific intervention characteristics were associated with variation in executive function outcomes [16]. Building on these earlier reviews, the present study provides an updated quantitative synthesis focused specifically on executive function and incorporates more recent randomized trials. In addition, where data permitted, we examined whether intervention characteristics were associated with variation in executive function outcomes. Subgroup analyses suggested more favorable pooled estimates for some protocols, such as walking-based or hourly interruption protocols; however, these findings were exploratory and should be interpreted cautiously because of the limited number of studies, small subgroup sizes, and low certainty of evidence.

The domain-specific findings showed that working memory improved significantly, whereas inhibitory control and cognitive flexibility did not reach statistical significance. One possible explanation is that several activity-break protocols included in this meta-analysis, such as walking and resistance-based activities, may engage motor execution and the maintenance of simple movement sequences, processes that could overlap with working-memory demands more directly than with task switching or conflict monitoring [57, 58]. Another possible explanation is that working memory may be relatively sensitive to short-term physiological changes induced by PA breaks, including increased arousal and changes in cerebral perfusion [18, 59, 60]. Previous studies have shown that sedentary behavior can reduce systemic circulation, which may be partly reversed by lower limb muscle pump activity [59]. Evidence from functional near-infrared spectroscopy has further indicated that short walking can increase oxygenated hemoglobin levels in the dorsolateral prefrontal cortex, potentially facilitating performance in working memory tasks [18, 60]. In contrast, the lack of significant effects on inhibitory control and cognitive flexibility may reflect their reliance on brain networks less immediately responsive to acute physiological changes. Inhibitory control is associated with sustained conflict monitoring via the anterior cingulate cortex–basal ganglia circuit [61], while cognitive flexibility engages the cerebellar-parietal network [62], both of which are more dependent on long-term neuroplasticity mediated by factors such as BDNF [63], rather than short-term hemodynamic shifts.

Regarding performance measures, PA breaks significantly improved RT but had no significant effect on accuracy. This finding is consistent with Garrett et al. [64], and suggests that PA may have a stronger acute effect on processing speed and response efficiency than on response precision. From the perspective of the drift-diffusion model, previous evidence suggest that PA-related cognitive advantages may be reflected more strongly in non-decision time than in decision threshold or drift rate [65]. This may indicate improvements in stimulus encoding or the motor response efficacy rather than changes in decision accuracy. Additionally, accuracy outcomes may be less sensitive to short-term changes, particularly when ceiling effects are present. Future studies should therefore report both speed- and accuracy-based outcomes and consider analytical approaches that distinguish processing speed from decision accuracy.

The exploratory moderator analyses suggested that PA breaks characteristics may contribute to heterogeneity in the observed effects. Low-intensity, walking-based breaks, and hourly interruption protocols showed favorable patterns in subgroup analyses. These findings are plausible, as light intensity activity may be sufficient to increase arousal without inducing fatigue [66, 67]. Walking was commonly used in the included studies and showed a favorable pooled estimate. Previous studies have suggested that walking breaks during sedentary behavior may benefit cardiometabolic and cognitive outcomes [18, 38, 42, 44, 48, 68, 69]. Walking may activate cerebellar-parietal pathways and enhance perceptual–motor integration, thereby indirectly supporting executive function [62]. However, given the low certainty of evidence and the absence of direct comparisons between activity types, these findings should be interpreted cautiously. Stair climbing also showed large effect sizes, but it was evaluated in only a small number of studies and therefor requires further validation. Similarly, although 60-minute interruption intervals appeared more favorable than more frequent interruptions, this finding should be interpreted cautiously. More frequent breaks may increase task-switching demands or cognitive load, but this explanation remains hypothetical. Moreover, evidence from cardiometabolic studies suggest that higher-frequency interruptions may be beneficial for other health outcomes [68–73], indicating that optimal interruption strategies may differ depending on the target outcomes. In addition, although age did not show a statistically significant moderating effect, this result should not be interpreted as evidence that age is unimportant. Most included studies involved relatively healthy adults, and the available evidence may have been underpowered to detect age-related differences. Older adults and individuals with lower baseline cognitive function or cognitive complaints may respond differently to PA breaks, given evidence linking sedentary behavior with cognitive function in older adults and experimental studies examining exercise or sitting-break effects on executive function in this population [19, 74]. These possibilities should be examined in future trials with broader age ranges and more detailed assessments of baseline cognitive status.

Several limitations should be acknowledged. First, the overall quality of evidence was low, primarily due to methodological limitations and imprecision in the included studies. Second, studies used acute laboratory-based crossover designs, which limits the generalizability of the findings to real-world and long-term settings [75]. Third, the physiological mechanisms underlying the observed effects remain uncertain. Future studies should incorporate neuroimaging tools such as functional near-infrared spectroscopy or functional magnetic resonance imaging to identify the neural pathways and physiological changes involved (e.g., activation in prefrontal-parietal networks, cerebral blood flow, neurotransmitter levels). Fourth, most studies included in this review did not differentiate effects based on population characteristics, such as age group or baseline cognitive status. Future research should extend to more diverse populations, including children, office workers, and older adults, and provide individualized recommendations. Finally, most of the studies included in this review employed walking, running, or resistance training, which place greater demands on working memory but relatively lower demands on inhibitory control and cognitive flexibility. Future studies incorporating task switching, dual-task elements, or inhibitory demands may help determine whether cognitively enriched PA produces broader executive function benefits.

## Conclusions

Despite the low-certainty evidence, this meta-analysis of existing studies suggests that interrupting prolonged sitting with short bouts of PA may yield small but significant improvements in executive function, especially in the working memory domain and RT performance. The effect is moderated by activity frequency, intensity, and type. These results provide quantitative evidence for optimizing interventions aimed at mitigating the cognitive impacts of prolonged sitting.

## Supplementary Information


Supplementary Material 1.



Supplementary Material 2.


## Data Availability

The datasets used and/or analysed during the current study are available from the corresponding author on reasonable request.

## Abbreviations

PA: Physical Activity
CI: Confidence Interval
BDNF: Brain-Derived Neurotrophic Factor
RT: Reaction Time
ACSM: American College of Sports Medicine
VO₂: Oxygen Uptake
%VO₂max: Percentage Of Maximal Oxygen Uptake
HR: Heart Rate
%HRmax: Percentage Of Maximal Heart Rate
HRR: Heart Rate Reserve
METs: Metabolic Equivalents
RPE: Rating Of Perceived Exertion
BMI: Body Mass Index
LPA: Light-intensity Physical Activity
MPA: Moderate-intensity Physical Activity
PI: Prediction intervals
RVE: Robust Variance Estimation
GRADE: Grading of Recommendations, Assessment, Development, and Evaluations
I^2^: I-squared statistic
PICO: Population, Intervention, Comparison, Outcome
PRISMA: Preferred Reporting Items for Systematic Reviews and Meta-Analyses
PROSPERO: International Prospective Register of Systematic Reviews
RoB: Risk-of-Bias tool
FITT-V: Frequency, Intensity, Type, Time, and Volume
