# Supplementary material for: Effect of breaking up prolonged sitting with physical activity on executive function: a three-level meta-analysis
Source: Int J Behav Nutr Phys Act. 2026 Jun 23;23:80. doi: 10.1186/s12966-026-01942-9 (PMC13536752; doi:10.1186/s12966-026-01942-9)
Supplement: Supplementary file 1 [file 12966_2026_1942_MOESM2_ESM.docx]

**Effect of** **Breaking Up Prolonged Sitting with Physical Activity on Executive Function: A Three-Level Meta-Analysis**

Supplementary Appendix

Table S1 PRISMA checklist

| **Section and Topic** | **Item #** | **Checklist item** | **Location where item is reported** |
| --- | --- | --- | --- |
| **TITLE** | | |  |
| Title | 1 | Identify the report as a systematic review. | 1 |
| **ABSTRACT** | | |  |
| Abstract | 2 | See the PRISMA 2020 for Abstracts checklist. | 1 |
| **INTRODUCTION** | | |  |
| Rationale | 3 | Describe the rationale for the review in the context of existing knowledge. | 2 |
| Objectives | 4 | Provide an explicit statement of the objective(s) or question(s) the review addresses. | 3 |
| **METHODS** | | |  |
| Eligibility criteria | 5 | Specify the inclusion and exclusion criteria for the review and how studies were grouped for the syntheses. | 3 |
| Information sources | 6 | Specify all databases, registers, websites, organisations, reference lists and other sources searched or consulted to identify studies. Specify the date when each source was last searched or consulted. | 3 |
| Search strategy | 7 | Present the full search strategies for all databases, registers and websites, including any filters and limits used. | 3 |
| Selection process | 8 | Specify the methods used to decide whether a study met the inclusion criteria of the review, including how many reviewers screened each record and each report retrieved, whether they worked independently, and if applicable, details of automation tools used in the process. | 4 |
| Data collection process | 9 | Specify the methods used to collect data from reports, including how many reviewers collected data from each report, whether they worked independently, any processes for obtaining or confirming data from study investigators, and if applicable, details of automation tools used in the process. | 4 |
| Data items | 10a | List and define all outcomes for which data were sought. Specify whether all results that were compatible with each outcome domain in each study were sought (e.g. for all measures, time points, analyses), and if not, the methods used to decide which results to collect. | 4 |
|  | 10b | List and define all other variables for which data were sought (e.g. participant and intervention characteristics, funding sources). Describe any assumptions made about any missing or unclear information. | 4 |
| Study risk of bias assessment | 11 | Specify the methods used to assess risk of bias in the included studies, including details of the tool(s) used, how many reviewers assessed each study and whether they worked independently, and if applicable, details of automation tools used in the process. | 4 |
| Effect measures | 12 | Specify for each outcome the effect measure(s) (e.g. risk ratio, mean difference) used in the synthesis or presentation of results. | 5 |
| Synthesis methods | 13a | Describe the processes used to decide which studies were eligible for each synthesis (e.g. tabulating the study intervention characteristics and comparing against the planned groups for each synthesis (item #5)). | 5 |
|  | 13b | Describe any methods required to prepare the data for presentation or synthesis, such as handling of missing summary statistics, or data conversions. | 5 |
|  | 13c | Describe any methods used to tabulate or visually display results of individual studies and syntheses. | 6 |
|  | 13d | Describe any methods used to synthesize results and provide a rationale for the choice(s). If meta-analysis was performed, describe the model(s), method(s) to identify the presence and extent of statistical heterogeneity, and software package(s) used. | 5 |
|  | 13e | Describe any methods used to explore possible causes of heterogeneity among study results (e.g. subgroup analysis, meta-regression). | 6 |
|  | 13f | Describe any sensitivity analyses conducted to assess robustness of the synthesized results. | 6 |
| Reporting bias assessment | 14 | Describe any methods used to assess risk of bias due to missing results in a synthesis (arising from reporting biases). | 6 |
| Certainty assessment | 15 | Describe any methods used to assess certainty (or confidence) in the body of evidence for an outcome. | 6 |
| **RESULTS** | | |  |
| Study selection | 16a | Describe the results of the search and selection process, from the number of records identified in the search to the number of studies included in the review, ideally using a flow diagram. | 7 |
|  | 16b | Cite studies that might appear to meet the inclusion criteria, but which were excluded, and explain why they were excluded. | 7 |
| Study characteristics | 17 | Cite each included study and present its characteristics. | 8 |
| Risk of bias in studies | 18 | Present assessments of risk of bias for each included study. | 11 |
| Results of individual studies | 19 | For all outcomes, present, for each study: (a) summary statistics for each group (where appropriate) and (b) an effect estimate and its precision (e.g. confidence/credible interval), ideally using structured tables or plots. | 13 |
| Results of syntheses | 20a | For each synthesis, briefly summarise the characteristics and risk of bias among contributing studies. | 12 |
|  | 20b | Present results of all statistical syntheses conducted. If meta-analysis was done, present for each the summary estimate and its precision (e.g. confidence/credible interval) and measures of statistical heterogeneity. If comparing groups, describe the direction of the effect. | 13 |
|  | 20c | Present results of all investigations of possible causes of heterogeneity among study results. | 14 |
|  | 20d | Present results of all sensitivity analyses conducted to assess the robustness of the synthesized results. | 15 |
| Reporting biases | 21 | Present assessments of risk of bias due to missing results (arising from reporting biases) for each synthesis assessed. | 16 |
| Certainty of evidence | 22 | Present assessments of certainty (or confidence) in the body of evidence for each outcome assessed. | 18 |
| **DISCUSSION** | | |  |
| Discussion | 23a | Provide a general interpretation of the results in the context of other evidence. | 19-22 |
|  | 23b | Discuss any limitations of the evidence included in the review. | 23 |
|  | 23c | Discuss any limitations of the review processes used. | 24 |
|  | 23d | Discuss implications of the results for practice, policy, and future research. | 22-23 |
| **OTHER INFORMATION** | | |  |
| Registration and protocol | 24a | Provide registration information for the review, including register name and registration number, or state that the review was not registered. | 3 |
|  | 24b | Indicate where the review protocol can be accessed, or state that a protocol was not prepared. | 3 |
|  | 24c | Describe and explain any amendments to information provided at registration or in the protocol. | 3 |
| Support | 25 | Describe sources of financial or non-financial support for the review, and the role of the funders or sponsors in the review. | 24 |
| Competing interests | 26 | Declare any competing interests of review authors. | 24 |
| Availability of data, code and other materials | 27 | Report which of the following are publicly available and where they can be found: template data collection forms; data extracted from included studies; data used for all analyses; analytic code; any other materials used in the review. | 24 |

*From:* Page MJ, McKenzie JE, Bossuyt PM, Boutron I, Hoffmann TC, Mulrow CD, et al. The PRISMA 2020 statement: an updated guideline for reporting systematic reviews. BMJ 2021;372:n71. doi: 10.1136/bmj.n71. This work is licensed under CC BY 4.0. To view a copy of this license, visit <https://creativecommons.org/licenses/by/4.0/>

Table S2 Search strategy

| Database | Search number | Query | Results |
| --- | --- | --- | --- |
| PubMed | #5 | #1 AND #2 AND #3 AND #4 | 423 |
|  | #4 | Break*[Title/Abstract] OR Interrupt*[Title/Abstract] OR Fraction*[Title/Abstract] OR Intersperse*[Title/Abstract] NOT Ejection Fraction[Title/Abstract] | 1,000,506 |
|  | #3 | Intervention[Title/Abstract] OR Trial[Title/Abstract] OR Random[Title/Abstract] OR RCT[Title/Abstract] OR Experiment*[Title/Abstract] | 4,459,364 |
|  | #2 | ("Cognition"[Mesh]) OR (Cognitions[Title/Abstract] OR "Cognitive Function"[Title/Abstract] OR "Cognitive Functions"[Title/Abstract] OR Function, Cognitive[Title/Abstract] OR Functions, Cognitive[Title/Abstract] OR "Cognitive Performance"[Title/Abstract] OR "Cognitive Ability"[Title/Abstract] OR "Executive Function"[Title/Abstract] OR Attention OR Memory) | 1,230,907 |
|  | #1 | ("Sedentary Behavior"[Mesh]) OR (Sedentary[Title/Abstract] OR Behavior, Sedentary[Title/Abstract] OR Sedentary Behaviors[Title/Abstract] OR Sedentary Lifestyle[Title/Abstract] OR Lifestyle, Sedentary[Title/Abstract] OR Physical Inactivity[Title/Abstract] OR Inactivity, Physical[Title/Abstract] OR Lack of Physical Activity[Title/Abstract] OR Sedentary Time[Title/Abstract] OR Sedentary Times[Title/Abstract] OR Time, Sedentary[Title/Abstract] OR Sitting[Title/Abstract]) | 82,464 |
| Web of Science | #1 | TS=(("sedentary behavior" OR "sedentary behaviour" OR sedentary OR "sedentary time" OR "sitting time" OR sitting OR "prolonged sitting" OR "uninterrupted sitting"))  AND TS=(break* OR "breaking up" OR interrupt* OR interruption* OR "physical activity break*" OR "activity break*" OR "exercise break*" OR "active break*" OR "walking break*" OR "standing break*" OR "sit-to-stand" OR "light-intensity walking" OR "moderate-intensity walking" OR cycling OR "activity bout*" OR intersperse* OR intermittent*)  AND TS=("executive function*" OR "cognitive function" OR "cognitive performance" OR "cognitive control" OR "inhibitory control" OR inhibition OR "working memory" OR "cognitive flexibility" OR "task switching" OR attention OR memory OR Stroop OR Flanker OR "Go/No-go" OR "go no go" OR "Trail Making" OR "reaction time")  AND TS=(random* OR trial* OR intervention* OR experiment* OR crossover OR "cross-over" OR acute OR controlled OR condition*) | 837 |
| Scopus | #1 | TITLE-ABS-KEY("sedentary behavior" OR "sedentary behaviour" OR sedentary OR "sedentary behaviors" OR "sedentary behaviours" OR "sedentary time" OR "sitting time" OR sitting OR "prolonged sitting" OR "uninterrupted sitting")  AND TITLE-ABS-KEY(break* OR "breaking up" OR interrupt* OR interruption* OR "physical activity break*" OR "activity break*" OR "exercise break*" OR "active break*" OR "walking break*" OR "standing break*" OR "sit-to-stand" OR "light-intensity walking" OR "moderate-intensity walking" OR cycling OR "activity bout*" OR intersperse* OR intermittent*)  AND TITLE-ABS-KEY("executive function*" OR "cognitive function" OR "cognitive performance" OR "cognitive control" OR "inhibitory control" OR inhibition OR "working memory" OR "cognitive flexibility" OR "task switching" OR attention OR memory OR Stroop OR Flanker OR "go no go" OR "Go/No-go" OR "Trail Making" OR "reaction time")  AND TITLE-ABS-KEY(random* OR trial* OR intervention* OR experiment* OR crossover OR "cross-over" OR acute OR controlled OR condition*) | 473 |
| EMBASE | #5 | #1 AND #2 AND #3 AND #4 | 1416 |
|  | #4 | 'randomized':ti,ab,kw OR 'randomised':ti,ab,kw OR 'random':ti,ab,kw OR 'trial':ti,ab,kw OR 'randomized controlled trial':it OR 'intervention*':ti,ab,kw OR 'experiment*':ti,ab,kw OR 'crossover':ti,ab,kw OR 'cross-over':ti,ab,kw OR 'acute':ti,ab,kw OR 'controlled':ti,ab,kw OR 'condition*':ti,ab,kw | 13030996 |
|  | #3 | 'executive function'/exp OR 'cognition'/exp OR 'executive function':ti,ab,kw OR 'executive functions':ti,ab,kw OR 'cognitive function':ti,ab,kw OR 'function cognitive':ti,ab,kw OR 'functions cognitive':ti,ab,kw OR 'cognitive performance':ti,ab,kw OR 'cognitive control':ti,ab,kw OR 'inhibitory control':ti,ab,kw OR 'inhibition':ti,ab,kw OR 'working memory':ti,ab,kw OR 'cognitive flexibility':ti,ab,kw OR 'task switching':ti,ab,kw OR 'stroop':ti,ab,kw OR 'flanker':ti,ab,kw OR 'go/no-go':ti,ab,kw OR 'trail making':ti,ab,kw OR 'reaction time':ti,ab,kw OR 'cognitive ability':ti,ab,kw OR 'attention':ti,ab,kw OR 'attentions':ti,ab,kw OR 'attentional':ti,ab,kw OR 'attentive':ti,ab,kw OR 'attentively':ti,ab,kw OR 'attentiveness':ti,ab,kw OR 'memories':ti,ab,kw OR 'memory':ti,ab,kw OR 'memorys':ti,ab,kw | 6030593 |
|  | #2 | 'break*':ti,ab,kw OR 'breaking up':ti,ab,kw OR 'interrupt*':ti,ab,kw OR 'interruption*':ti,ab,kw OR 'physical activity break*':ti,ab,kw OR 'activity break*':ti,ab,kw OR 'exercise break*':ti,ab,kw OR 'exercise snack*':ti,ab,kw OR 'accumulated exercise*':ti,ab,kw OR 'active break*':ti,ab,kw OR 'walking break*':ti,ab,kw OR 'standing break*':ti,ab,kw OR 'sit-to-stand':ti,ab,kw OR 'light-intensity walking':ti,ab,kw OR 'moderate-intensity walking':ti,ab,kw OR 'cycling':ti,ab,kw OR 'activity bout*':ti,ab,kw OR 'intersperse*':ti,ab,kw OR 'intermittent*':ti,ab,kw OR 'fraction*':ti,ab,kw | 1889272 |
|  | #1 | 'sedentary lifestyle'/exp OR 'sedentary lifestyle' OR 'sedentary':ti,ab,kw OR 'sedentary behavior':ti,ab,kw OR 'behavior sedentary':ti,ab,kw OR 'sedentary behaviour':ti,ab,kw OR 'sedentary behaviors':ti,ab,kw OR 'sedentary behaviours':ti,ab,kw OR 'sedentary lifestyle':ti,ab,kw OR 'lifestyle sedentary':ti,ab,kw OR 'physical inactivity':ti,ab,kw OR 'inactivity physical':ti,ab,kw OR 'lack of physical activity':ti,ab,kw OR 'sedentary time':ti,ab,kw OR 'sedentary times':ti,ab,kw OR 'time sedentary':ti,ab,kw OR 'sitting time':ti,ab,kw OR 'sitting':ti,ab,kw OR 'prolonged sitting':ti,ab,kw OR 'uninterrupted sitting':ti,ab,kw OR 'classroom sitting':ti,ab,kw OR 'workplace sitting':ti,ab,kw | 136039 |
| Cochrane Library | #1 | MeSH descriptor: [Sedentary Behavior] explode all trees | 2150 |
|  | #2 | (sedentary OR "sedentary behavior" OR "sedentary behaviour" OR "sedentary time" OR "sitting time" OR sitting OR "prolonged sitting" OR "uninterrupted sitting") | 23924 |
|  | #3 | (break* OR "breaking up" OR interrupt* OR interruption* OR physical activity NEXT break* OR activity NEXT break* OR exercise NEXT break* OR active NEXT break* OR walking NEXT break* OR standing NEXT break* OR "sit-to-stand" OR "light-intensity walking" OR "moderate-intensity walking" OR cycling OR activity NEXT bout* OR intersperse* OR intermittent*) | 75228 |
|  | #4 | MeSH descriptor: [Executive Function] explode all trees | 2102 |
|  | #5 | (executive NEXT function* OR "cognitive function" OR "cognitive performance" OR "cognitive control" OR "inhibitory control" OR inhibition OR "working memory" OR "cognitive flexibility" OR "task switching" OR attention OR memory OR Stroop OR Flanker OR "Go/No-go" OR "go no go" OR "Trail Making" OR "reaction time") | 139146 |
|  | #6 | (random* OR trial* OR intervention* OR experiment* OR crossover OR "cross-over"  OR acute OR controlled OR condition*) | 2388129 |
|  | #7 | (#1 OR #2) AND #3 AND (#4 OR #5) AND #6 | 544 |
| APA PsycINFO | #1 | (sedentary OR "sedentary behavior" OR "sedentary behaviour" OR "sedentary time" OR "sitting time" OR sitting OR "prolonged sitting" OR "uninterrupted sitting") AND (break* OR "breaking up" OR interrupt* OR interruption* OR "physical activity break*" OR "activity break*" OR "exercise break*" OR "active break*" OR "walking break*" OR "standing break*" OR "sit-to-stand" OR intermittent*) AND ("executive function*" OR "cognitive function" OR "cognitive performance" OR "inhibitory control" OR inhibition OR "working memory" OR "cognitive flexibility" OR attention OR memory OR Stroop OR Flanker OR "Go/No-go" OR "Trail Making" OR "reaction time") AND (random* OR trial* OR intervention* OR experiment* OR crossover OR "cross-over" OR acute OR controlled OR condition*) | 70 |
| SPORTDiscus | #1 | (sedentary OR "sedentary behavior" OR "sedentary behaviour" OR "sedentary time" OR "sitting time" OR sitting OR "prolonged sitting" OR "uninterrupted sitting") AND (break* OR "breaking up" OR interrupt* OR interruption* OR "physical activity break*" OR "activity break*" OR "exercise break*" OR "active break*" OR "walking break*" OR "standing break*" OR "sit-to-stand" OR intermittent*) AND ("executive function*" OR "cognitive function" OR "cognitive performance" OR "inhibitory control" OR inhibition OR "working memory" OR "cognitive flexibility" OR attention OR memory OR Stroop OR Flanker OR "Go/No-go" OR "Trail Making" OR "reaction time") AND (random* OR trial* OR intervention* OR experiment* OR crossover OR "cross-over" OR acute OR controlled OR condition*) | 45 |

**Table S3. Detailed Variable Coding**

| Category | Variable | Coding Description | Levels |
| --- | --- | --- | --- |
| Executive Function Subdomains | EF Domain | Based on classical three-dimensional model[1] | 1. Working memory  2. Inhibitory control  3. Cognitive flexibility |
| Task Performance Indicators | Outcome Type | performance metrics | 1. Accuracy (positive-oriented) 2. Reaction Time (RT, reverse-coded) |
| PA Intervention Parameters |  |  |  |
|  | Interruption Mode | Pattern of physical activity breaks | Studies were eligible only if prolonged sitting was interrupted by multiple physical activity bouts; single-bout interventions were not included in the final synthesis. |
|  | Intensity | Exercise intensity level. Classified using author-reported intensity; if unavailable, METs, HRR, VO₂ reserve, HRmax, RPE, workload, walking speed, or intervention description were used. Oxygen uptake-based indicators were prioritized over heart-rate indicators when both were available. These thresholds were based on established exercise-intensity classification criteria from the American College of Sports Medicine and commonly used adult physical activity intensity definitions[2]. | Light-intensity physical activity: 1.6–2.9 METs; <40% HRR or VO₂ reserve; <64% HRmax; Borg RPE 9–11 on the Borg 6–20 scale; or low-effort activity description. 2. Moderate-intensity physical activity: 3.0–5.9 METs; 40–59% HRR or VO₂ reserve; 64–76% HRmax; Borg RPE 12–13 on the Borg 6–20 scale; or moderate-effort activity description. 3. Vigorous- or high-intensity physical activity: **≥6.0 METs, ≥60% heart rate reserve or VO₂ reserve, ≥77% maximal heart rate, or RPE ≥14 on the Borg 6–20 scale**. |
|  | Type | Specific activity type | 1. Walking 2. Stair climbing 3. Resistance training 4. Cycling 5. Yoga |
|  | Frequency | Timing of intervention sessions | 1. Once per hour 2. Twice per hour 3. More per hour |
|  | Duration | Length of each session | 1. 1-2 minutes 2. 3-5 minutes 3. ≥6 minutes |
| Participant Characteristics |  |  |  |
|  | Age Group | Developmental/life stage categories | 1. Adolescents (Ages 13-17 years) 2. Young adults (Ages 18-34 years) 3. Middle-aged (Ages 35-64 years) 4. Older adults (Ages ≥65 years) |
|  | Gender Composition | Sex distribution in sample | 1. Female-only 2. Male-only 3. Mixed |
|  | Weight Status | Body mass classification following World Health Organization classification standards[3] | 1. Normal BMI (BMI ranging from 18.5 to 24.9 kg/m² for adults) 2. Overweight/Obesity (overweight was defined as BMI 25.0-29.9 kg/m² and obesity as BMI ≥30.0 kg/m² for adults) |

Figure S1 Influential case diagnosis

Figure S2 Leave-one-out sensitive analysis

Table S4 Leave-one-out sensitive analysis

| Study | estimate | lowerCI | upperCI | lowerPI | upperPI |
| --- | --- | --- | --- | --- | --- |
| Mullane et al. 2017 | 0.110 | 0.012 | 0.209 | -0.233 | 0.453 |
| Bergouignan et al. 2016 | 0.134 | 0.028 | 0.240 | -0.249 | 0.517 |
| Wanders et al. 2020 | 0.126 | 0.020 | 0.233 | -0.262 | 0.515 |
| Wu et al. 2023 | 0.127 | 0.021 | 0.233 | -0.260 | 0.514 |
| Duvivier et al. 2024 | 0.129 | 0.024 | 0.233 | -0.253 | 0.511 |
| Pindus et al. 2025 | 0.130 | 0.023 | 0.237 | -0.259 | 0.519 |
| Kuang et al. 2025 | 0.141 | 0.040 | 0.241 | -0.216 | 0.497 |
| Chandran et al. 2023 | 0.102 | 0.020 | 0.184 | -0.144 | 0.348 |
| Chrismas et al. 2019 | 0.134 | 0.031 | 0.237 | -0.239 | 0.507 |
| Heiland et al. 2021 | 0.134 | 0.027 | 0.240 | -0.251 | 0.519 |
| Wennberg et al. 2016 | 0.123 | 0.020 | 0.225 | -0.251 | 0.497 |
| Wheeler et al. 2019 | 0.115 | 0.010 | 0.219 | -0.258 | 0.487 |
| Kjellenberg et al. 2024 | 0.113 | 0.010 | 0.217 | -0.254 | 0.481 |
| Chueh et al. 2025 | 0.125 | 0.020 | 0.231 | -0.261 | 0.511 |
| Maasakkers et al. 2020 | 0.131 | 0.024 | 0.237 | -0.257 | 0.518 |
| da Silva et al. 2024 | 0.132 | 0.025 | 0.239 | -0.257 | 0.520 |
| Kuo et al. 2024 | 0.151 | 0.062 | 0.239 | -0.138 | 0.440 |
| Horiuchi et al. 2023 | 0.117 | 0.013 | 0.222 | -0.259 | 0.494 |
| Cunha et al. 2025 | 0.125 | 0.018 | 0.231 | -0.264 | 0.514 |
| Charlett et al. 2021 | 0.126 | 0.024 | 0.228 | -0.247 | 0.500 |
| Kamath et al. 2025 | 0.130 | 0.024 | 0.237 | -0.257 | 0.518 |
| Stoner et al. 2019 | 0.133 | 0.030 | 0.236 | -0.241 | 0.507 |

Figure S3 Forest plot (with influential cases)

Figure S4 Forest plot (without influential cases)
